# The FITNESS study: longitudinal geriatric assessment, treatment toxicity, and biospecimen collection to assess functional disability among older adults with lung cancer

**DOI:** 10.3389/fragi.2024.1268232

**Published:** 2024-06-07

**Authors:** Madison Grogan, Rebecca Hoyd, Jason Benedict, Sarah Janse, Nyelia Williams, Michelle Naughton, Christin E. Burd, Electra D. Paskett, Ashley Rosko, Daniel J. Spakowicz, Carolyn J. Presley

**Affiliations:** ^1^ Division of Medical Oncology, Department of Internal Medicine, The Ohio State University, Columbus, OH, United States; ^2^ Center for Biostatistics, The Ohio State University Wexner Medical Center, Columbus, OH, United States; ^3^ Division of Cancer Prevention and Control, Department of Medicine, College of Medicine, and The Ohio State University Comprehensive Cancer Center, The Ohio State University Columbus, OH, United States; ^4^ Pelotonia Institute for Immuno-Oncology, The Ohio State University James Comprehensive Cancer Center–James Cancer Hospital and Solove Research Institute, Columbus, OH, United States; ^5^ Departments of Molecular Genetics, Cancer Biology, and Genetics, The Ohio State University Comprehensive Cancer Center, Columbus, OH, United States

**Keywords:** lung cancer, functional decline, aging, biomarkers, microbiome, novel treatments

## Abstract

**Introduction:**

Older adults with chronic disease prioritize functional independence. We aimed to describe the feasibility of capturing functional disability and treatment toxicity among older adults with lung cancer using a longitudinal comprehensive geriatric assessment (CGA) and molecular biomarkers of aging.

**Methods:**

This prospective study included adults ≥60 years with any newly diagnosed non-small-cell lung cancer. Participants were recruited from central Ohio (2018–2020). Study assessments included the Cancer and Aging Research Group CGA (CARG-CGA), short physical performance battery (SPPB), and the blessed orientation-memory concentration (BOMC) test at baseline, 3, 6, and 12 months. Activities of daily living (ADLs) and instrumental ADLs (IADLs), quality of life (QoL, PROMIS 10), and treatment toxicity were captured monthly. Stool and blood were collected to characterize the gut microbiome and age-related blood biomarkers.

**Results:**

This study enrolled 50 participants with an average age of 71.7 years. Ninety-two percent of participants were Caucasian, 58% were male, and all were non-Hispanic. Most had advanced stage (stage III/IV: 90%; stage I/II: 10%), with adenocarcinoma the predominant histologic subtype (68% vs. 24% squamous). First-line treatments included chemotherapy (44%), immune checkpoint inhibitors (ICIs, 22%), chemotherapy and ICIs (30%), or tyrosine kinase inhibitors (4%). The median baseline CARG toxicity score was 8 (range 2–12). Among patients with treatment-related toxicity (*n* = 49), 39 (79.6%) cases were mild (grade 1–2), and 10 (20.4%) were moderate to severe (≥ grade 3). Treatment toxicity was greater among those with a CARG score ≥8 (28.0% vs. 13.6%). Higher IADL independence, QoL, and SPPB scores at baseline were positively associated with *Candidatus Gastranaerophilales bacterium*, *Lactobacillus rogosae*, and *Enterobacteria phage P4. Romboutsia ilealis*, *Streptococcus*, and *Lachnoclostridium sp An138* and T cell *lag3* and *cd8a* were associated with worse IADLs, QoL, and SPPB scores at baseline.

**Discussion:**

A longitudinal CGA and biomarker collection is feasible among older adults undergoing lung cancer treatment. Gut microbe and T cell gene expression changes correlated with subjective and objective functional status assessments. Future research will test causality in these associations to improve outcomes through novel supportive care interventions to prevent functional disability.

## 1 Introduction

Lung cancer is the leading cause of cancer-related death in the United States (United States) and is primarily a disease of older adults with a median age of 71 years at diagnosis (Bade and Dela Cruz, 2020). This is critical as older adults are the fastest-growing demographic in the United States, projected to make up almost two-thirds of all cancer diagnoses by 2030 (Smith et al., 2009; Hurria et al., 2013). Despite this knowledge, clinical trials primarily include younger, healthier adults experiencing cancer, leaving clinicians, patients, and families uncertain about treatment-related toxicity, disease response, and functional status (Vora and Reckamp, 2008).

Novel cancer drugs, such as immune checkpoint inhibitors (ICIs) and oral tyrosine kinase inhibitors (TKIs), have dramatically shifted the treatment course for advanced and early-stage non-small-cell lung cancer (NSCLC), further complicating the prediction of treatment tolerability among older adults. However, there is evolving evidence that predictive biomarkers and a comprehensive geriatric assessment (CGA) can identify older adults likely to benefit from cancer treatment, experience toxicity (Corre et al., 2016), suffer functional decline (Presley et al., 2022), and experience worse quality of life (Gomes et al., 2021; Presley et al., 2021). Past research typically involves a single CGA and subsequent outcomes assessments. The feasibility of a longitudinal assessment with repeated measures is unknown. Understanding how GA measures change over time and are associated with treatment toxicity and functional status is critical. Most older adults with serious illnesses, such as advanced cancer, prioritize remaining functionally independent over prolonged survival (Fried et al., 2002; Osoba et al., 2006; Li et al., 2022).

Functional decline and worsening disability among older adults with cancer are typically associated with increased mortality, loss of independent living, and substantial financial costs (Fried and Guralnik, 1997; Guralnik et al., 2002; Fong, 2019). Prior assessment tools, including the Eastern Cooperative Oncology Group (ECOG) performance status, were designed to predict toxicity; but, these tools were developed among patients receiving chemotherapy and not newer treatments such as ICIs and TKIs (Oken et al., 1982; Repetto et al., 2002; Hurria et al., 2011; Extermann et al., 2012; Corre et al., 2016). This is important because even a single cycle of chemotherapy can result in functional decline, depression, and low instrumental activities of daily living (IADL) scores in this vulnerable group (Hoppe et al., 2013). Furthermore, up to 40% of patients with advanced NSCLC experience functional disability at diagnosis (Presley et al., 2016). These facts underscore the need for an improved understanding of the relationship between functional status, treatment outcomes, and survival among patients with lung cancer receiving modern systemic treatments such as ICIs and TKIs (Wong et al., 2022).

The concomitant collection of aging biomarkers has also been absent in previous work. Biological aging of the immune system could impact tumor response to ICIs. Therefore, developing non-invasive tests to understand immune changes during lung cancer treatment is urgently needed. The aging immune system could mean disproportionate rates of autoimmune inflammatory conditions that can occur with ICI treatment, such as rash, thyroiditis, pneumonitis, colitis, arthritis, or hepatitis (Atchley et al., 2021). The gut microbiome is the one biomarker and therapeutic target that can both predict treatment toxicities and is modifiable to improve disease response. Recent findings suggest that certain microbes in the gut modulate the outcome of ICI treatments (Pitt et al., 2017; Routy et al., 2018). The quantification and characterization of immune changes in peripheral blood over time, in conjunction with changes in the gut microbiome, may provide information on how patients are likely to respond to a given treatment. The first step in examining this relationship among humans with solid tumors is longitudinal biospecimen collection and analysis to characterize and describe the diversity among older adults with lung cancer.

This study aimed to 1) test the feasibility of the Cancer and Aging Research Group (CARG) longitudinal CGA, objective physical function and cognitive evaluation, monthly patient-reported outcomes (PROs), and concomitant biospecimen collection among patients with newly diagnosed NSCLC and 2) evaluate associations between the CARG-CGA assessment scores, treatment toxicity, and changes in the gut microbiome and peripheral blood aging biomarkers.

## 2 Methods

### 2.1 Study design

This was a pilot prospective longitudinal cohort study of 50 adults ≥60 years of age with any stage of untreated or recurrent NSCLC. Eligible participants underwent a baseline assessment using the CARG CGA (Hurria et al., 2005; Nie et al., 2013). Briefly, this assessment included the CARG chemotherapy toxicity calculator, the Patient-Reported Outcome Measurement Information System (PROMIS 10 (Hays et al., 2009) Global Health Scale Short Form v1.1, and sociodemographic information. The CARG CGA includes self-reported functional status measures, comorbidity, medications, nutrition, psychological state, and social support. A clinical research coordinator administered the cognitive assessment, the Blessed Orientation-Memory-Concentration test (BOMC test (Tuch et al., 2021)), and the short physical performance battery (SPPB) (Guralnik et al., 1994; Kawas et al., 1995). Participants also underwent longitudinal peripheral blood draws and stool sample collection. Participants then received standard-of-care lung cancer treatment or participated in a clinical trial as decided by their treating physician.

### 2.2 Eligibility criteria

Participants were recruited, consented, and enrolled in the FITNESS study at The Ohio State University Comprehensive Cancer Center (OSUCCC)–James Thoracic Oncology clinic. Eligible patients were ≥60 years of age and intended to receive systemic treatment at the OSUCCC for either a new or recurrent NSCLC diagnosis. Participants were required to have decision-making capacity and willingness to sign informed consent. Patients with any medical conditions, including but not limited to unstable angina pectoris, cardiac arrhythmia, psychiatric illness, or cognitive impairment, without a legally authorized representative (LAR) that would verify compliance with study procedures were excluded.

### 2.3 Study procedures

Participants underwent a baseline assessment before initiating lung cancer treatment. This process included sociodemographic and disease assessment; CARG toxicity tool calculation (Hurria et al., 2011); stool and blood sample collection; and completion of the CARG CGA, the BOMC, the SPPB (Almugbel et al., 2022), and the timed up-and-go (TUG) (Nakano et al., 2021).

The type of treatment and drug doses (planned and actual) were recorded along with any supportive care medications given throughout treatment. Grade 1–5 toxicities (National Cancer Institute Common Terminology Criteria for Adverse Events, NCI CTCAE V5) (Kluetz et al., 2016), hospitalizations, dose delays and reductions, and drug discontinuation were captured. The toxicity assessment and chemotherapy drug and dose information were collected by a study coordinator at the beginning of every treatment cycle, reviewed and attributed by two certified oncologists. Vital signs, labs, and health-related quality of life questionnaires, including the PROMIS 10^28^ and European Organization for Research and Treatment of Cancer (EORTC) Quality of Life Questionnaire for Lung Cancer (QLQ-LC-13) (Bergman et al., 1994), were collected at visit 2 (day 90) and visit 3 (day 180). Patients underwent additional monthly functional status assessments (FSA), monthly biospecimen banking, and an additional CGA at visit 3.

Baseline assessments were repeated at the end-of-treatment (EOT) visit. These occurred up to 45 days after completing treatment or 365 days after treatment initiation—whichever occurred first. Due to the varying length of treatment plans and different staging at the time of diagnosis, participants prescribed only four cycles of chemotherapy (adjuvant treatment) were only eligible to participate in the baseline and EOT visits. Visit 1 (baseline), visit 2 (day 90), and visit 3 (day 180) allowed a ±30-day window of time, letting patients enroll up to 30 days after treatment initiation. Disease response was calculated using Response Evaluation Criteria in Solid Tumors (RECIST) 1.1 criteria by two medical oncology fellows using chest cat scan (CT) imaging (Eisenhauer et al., 2009). Target lesions were followed throughout the study at each visit in which imaging was available. Tumor response was categorized into four groups: complete response, partial response, stable disease, and progressive disease.

Stool samples were collected at baseline and during participants’ oncology visits, roughly every 3–4 weeks. Patients were provided with a stool collection tub and collection instructions. Patients were asked to collect a sample within 24 h of returning to the clinic. Once the biospecimen sample reached the lab, it was aliquoted into cryovials and stored at −80°C. Samples were then processed in batches. DNA was extracted using the QIAGEN Power Fecal Pro kit. Sequencing was performed on the Illumina NovaSeq 6,000 platform to a depth of eight million reads per sample. Demultiplexed fastq files were cleaned, paired, and taxonomically assigned following the MetaPhlAn HUMAnN 3 Pipeline (Beghini et al., 2021).

Peripheral blood (40 mL) was collected at baseline, 6 months, and at either 1 year or the end of the patient’s treatment plan. Blood samples were only collected from participants who had not initiated treatment before visit one to control the purity of data. The samples were processed by The Ohio State University Leukemia Tissue Bank (LTB). Peripheral blood T cells were isolated *via* negative selection using the RosetteSep human T cell isolation cocktail (Stemcell Technologies). Tcell RNA was isolated using a Zymo Research quick-DNA/RNA miniprep kit. The quality and concentration of the resulting RNA was quantified on an Agilent TapeStation and analyzed using a custom NanoString codeset panel, OSU Senescence. The OSU Senescence panel is comprised of 74- T-cell markers with five loading controls. Gene expression markers were selected to measure subset-specific T-cell mRNA markers of cellular senescence and T-cell differentiation proliferation, and exhaustion as previously published (Rosko et al., 2019; Lucas et al., 2020; Fell et al., 2022). Additionally, certain transcription profiles in T cells such as LAG3 and CD8 transcription expression has been shown to be associated with inferior clinical outcomes in patients with myeloma (Lucas et al., 2020). T cells are exhausted when cells lose cytotoxic capacity, show sustained expression of inhibitory checkpoint molecules such as LAG3 and have distinct altered transcription profiles in CD8 subsets (Lucas et al., 2020). Therefore, LAG3 and CD8 transcription profiles were examined for associations with longitudinal IADL scores.

### 2.4 Statistical analysis

The primary aim of this study was to assess the feasibility of a longitudinal geriatric assessment collected concurrently with blood and stool biomarkers. Metrics included the number of blood and stool samples collected, the completion of PRO assessments, and patient withdrawal. Feasibility was defined *a priori* as the proportion of missing information at each time point, with success defined as at least 50% of participants able to complete study visits 2, 3, and four or EOT. Reasons for missed collections and study withdrawals were captured.

Descriptive statistics were used to summarize demographic information, with totals and percentages used for categorical data and medians and interquartile ratio (IQR) used for continuous data. When determining the immune-related adverse event (irAE) grade for a given time point, the grade of the most extreme irAE for that time point was used. For association with baseline CARG, the maximum-grade irAE experienced by the patient was considered.

Microbe and gene expression associations with the CGA were performed using longitudinal mixed-effects models using the {lme4} and {lmerTest} packages in R (Bates et al., 2015). A model was run for each combination of GA tool (i.e., SPPB or IADL), microbe, and gene. For associations between microbes and the selected GA tool, each GA tool was considered an independent variable and all other variables were considered dependent variables. Repeated measures in patients were handled using random effects for each patient. All other effects were fixed. The longitudinal nature of the data was accounted for by including a term for the number of days on the study associated with each measure. The models can be described by the following equation, in which the term *A*_*i*_ is the score (*A*) for each geriatric assessment(i), *M*_*j*_ is the relative abundance (*M*) for each microbe *(j), D* is days on study, *C* is immune checkpoint inhibitor, (1|*P*_*k*_) is the intercept (1|*P)* for each patient *(k):*

$$A_{i,k}=M_{j,k}+D+C+\left(1|P_{k}\right)$$

For transcript and geriatric assessment tools, variables were centered and scaled before modeling. Geriatric assessments remained the independent variable and patient intercepts remain random effects. The effects of change over time are still controlled for by including days on study in the model. These models can be described with the following equation, where *A*_*i*_ is the score *(A)* geriatric assessment of interest *(i), G*_*j*_ is the expression *(G)* of the gene of interest *(i), D* is days on study, (1|*P*_*k*_) is the intercept (1|*P)* for each patient *(k):*

$$A_{i,k}=G_{j,k}+D+\left(1|P_{k}\right)$$

Analyses were performed in R, version 4.1.

## 3 Results

### 3.1 Feasibility

**FIGURE 1:**
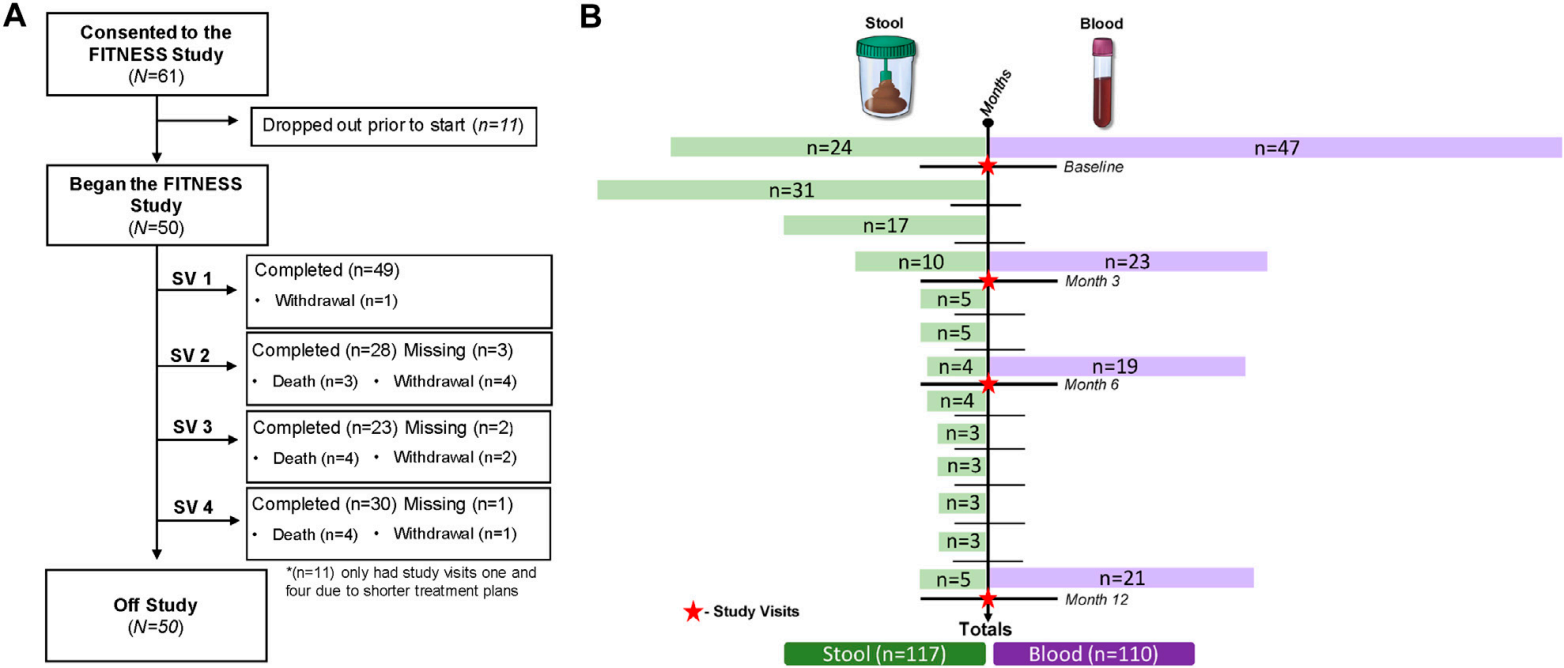
Study enrollment, follow-up, dropout, and biomarker collection. **(A)** CONSORT diagram; **(B)** longitudinal biomarker collection.

The study enrolled 61 patients with 11 withdrawals prior to starting (18% dropout rate), resulting in 50 evaluable participants at baseline. Among the 11 patients who dropped out before the study began, the reasons for withdrawal included disease progression, declining performance status, unwillingness to complete the study assessments, and study burden, including increased visit time and survey fatigue. Among the 50 participants, one withdrew at visit one due to cognitive impairment without an LAR. Reasons for drop out prior to the 3-month time point were death (*n* = 3) and withdrawal (*n* = 4). Inclusion criteria required participants to be undergoing active treatment *versus* surveillance for longitudinal follow up; therefore, the three- and 6-month time points excluded an additional 11 participants as they had completed treatment (adjuvant chemotherapy). Excluding the 11 adjuvant chemotherapy participants, at 3-months, 28 of 31 eligible participants (90%) completed the visit two assessments. Excluding the 11 adjuvant chemotherapy patients, at 6 months, 23 of 25 eligible participants (92%) had completed the study assessments (study visit 3). Reasons for drop out prior to 6 months were death (*n* = 4) and withdrawal (*n* = 2). For study visit 4, 30 (97%) of the remaining 31 participants completed the assessments. Reasons for drop out prior to 1-year were death (*n* = 4) and withdrawal (*n* = 1). The overall study attrition rate was 38%, excluding those who wished to continue participation after refusing to complete at least one study visit. Common reasons for study drop out included death (22%), loss of interest in participation (14%), and cognitive impairment (2%)—The study CONSORT diagram in Figure 1A details study enrollment, follow-up, and dropout.

The collection of longitudinal gut microbiome samples posed challenges, including patient hesitancy, transportation difficulties for those with longer travel times, and constipation before the return-to-clinic visit. Despite these barriers, 113 stool samples were collected, including 24 baseline (treatment-naive) specimens. Of those, 23 (95.8%) participants provided longitudinal samples for correlative analysis. See Figure 1B for a detailed overview of longitudinal biomarker collection.

### 3.2 Patient characteristics

**FIGURE 2:**
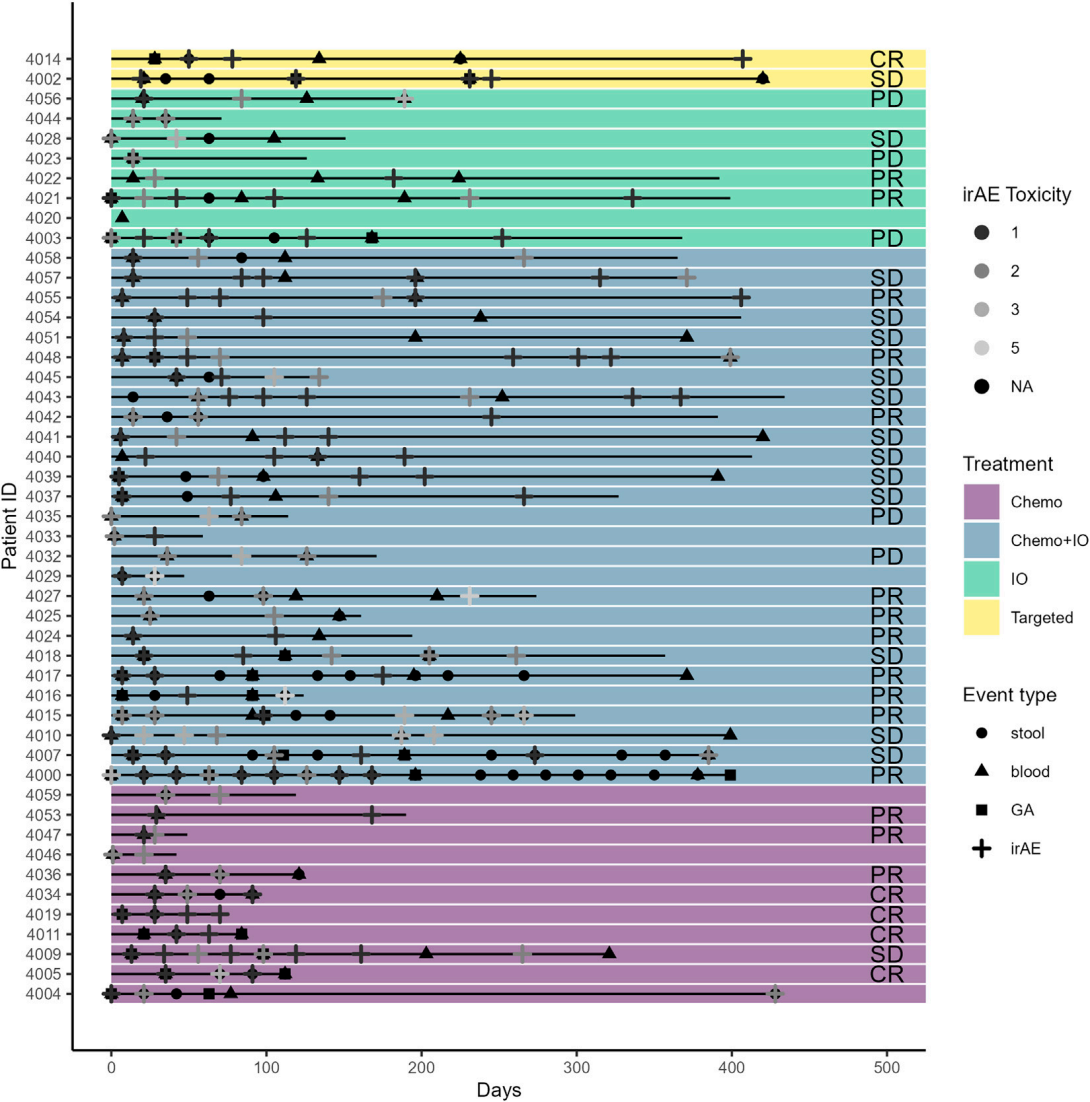
Treatment type, toxicity, geriatric assessment, and biospecimen timeline for study participants.

The mean age at baseline was 71.7 years (60–88 years). Most participants were Caucasian (92%), and 58% of patients were male. The most common histology was adenocarcinoma (70%), followed by squamous cell carcinoma (24%). Most cases were advanced stage (90% stage III/IV). For first-line treatment, 44% of patients received chemotherapy, 30% received a combination of chemotherapy and ICIs, 22% received ICIs alone, and 2 (4%) received TKIs. The mean CARG chemotherapy toxicity score at baseline was 7.5 (range 2–12), indicating an intermediate risk of treatment toxicity. Patient demographics and characteristics are further detailed in Table 1. Patient treatment timelines with the treatment types, treatment responses, and events, including toxicities and biomarker obtainment, are displayed in Figure 2.

**TABLE 1:** Patient characteristics *N* = 50.

Characteristic	n (%)
Age (years)	
60-69	17 (34)
70-79	29 (58)
80+	4 (8)
Sex	
Male	29 (58)
Female	21 (42)
Ancestry	
White	46 (92)
Black or African American	2 (4)
Asian	1 (2)
Unknown/not reported	1 (2)
Cancer Type	
Adenocarcinoma	35 (70)
Squamous	12 (24)
Adenosquamous	0 (0)
Not Otherwise Specified (NOS)	3 (6)
Cancer Stage	
I/II	5 (10)
III/IV	45 (90)
First-Line Treatment Category	
Chemotherapy	21 (44)
Combination Chemotherapy + ICIs	16 (30)
ICIs Alone	11 (22)
Tyrosine Kinase Inhibitors	2 (4)

* Abbreviations: ICI, immune checkpoint inhibitors

### 3.3 Comprehensive geriatric assessment and patient-reported outcomes

The CARG-CGA subset on IADLs (median: 14), Medical Outcomes Study (MOS) Physical Health (median: 61), Self-report Health Rating (median: 90), and the MOS Mental Health Inventory (median: 81) indicate relatively high levels of independence and mental health statuses among this cohort at baseline. Similarly, the PROMIS 10 average functional disability score (median: 24) and the EORTC-LC subset score (median: 17) indicated minimal to no baseline disability and minimal levels of symptom burden (Table 2; complete results including longitudinal data appear in Supplementary Table S4). While participants scored highly on the Social Support Medical Outcomes Survey (median: 96), their scores demonstrated a moderate impact to their usual levels of social activities (median: 58). The CARG-CGA Comorbidity Index showed that participants had an average of six pre-existing comorbid conditions present at baseline.

**TABLE 2:** Comprehensive geriatric assessment at baseline.

Instrument	n (%)	Median (IQR)
**PROMIS 10**	45 (90)	34 (29, 40)
**EORTC-QLQ-LC-13**	44 (88)	17 (11, 20)
**FSA**	44 (88)	
13-item		0 (0, 1)
7-item		0 (0, 0)
**CARG-GA**	47 (94)	
Instrumental Activities of Daily Living (IADL)		14 (13, 14)
Medical Outcomes Study (MOS) Physical Health		61 (39, 78)
Self-report Health Rating		90 (80,95)
Comorbidity: Physical Health Section		6 (4, 7)
Psychological Status: MHI		82 (69, 91)
Social Functioning: Medical Outcomes Activity Limitations Measure		58 (42, 67)
Social Support: Medical Outcomes Social Support Survey		96 (80, 100)
Religiosity		18 (13, 23)
**TUG**	43 (86)	
No Fall Risk	24 (48)	
Mild Fall Risk	19 (38)	
**BOMC**	43 (88)	6 (2, 8)
**SPPB**	45 (90)	9 (7.3, 11)
	**CARG Toxicity Score n (row%)**	
**Highest Grade Toxicity**	<7	8+
**1-2**	19 (51.4)	18 (48.7)
**3-5**	3 (30)	7 (70)
**Total**	22	25

* Abbreviations: PROMIS 10, Patient-Reported Outcome Measurement Information System; CARG, Cancer and Aging Research—Geriatric Assessment; Group FSA, Functional Status Assessment; EORTC-LC-13, European Health-Related Quality of Life Lung Cancer Subset; MHI, Mental Health Index; TUG, Timed Up and Go; Blessed Orientation Memory Concentration, BOMC; SPPB, Short Physical Performance Battery

**FIGURE 3:**
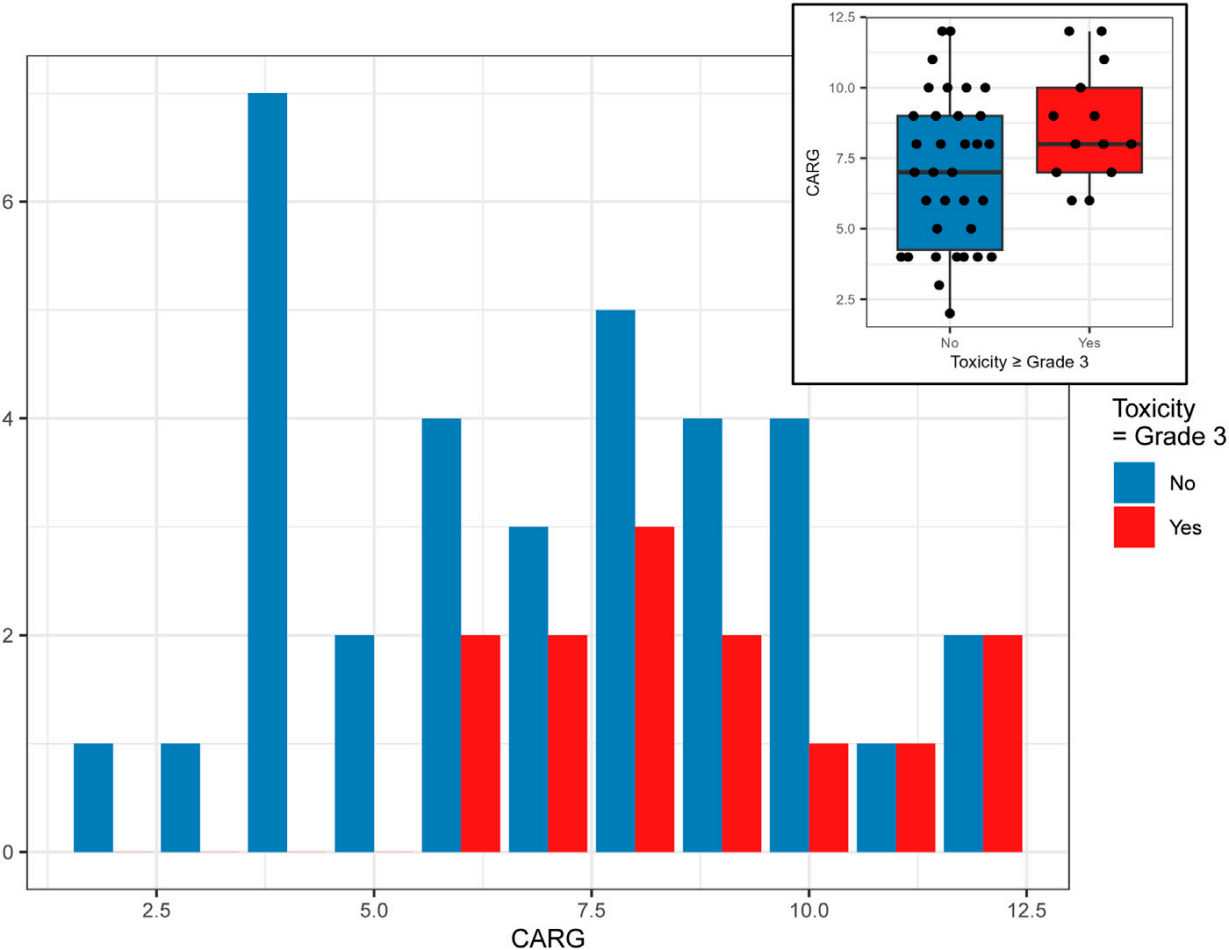
Prediction of immune-related adverse events using baseline CARG and longitudinal GAs.

Using the CARG chemotherapy toxicity tool, 22 patients (47%) had a low or mild risk of chemotherapy toxicity, and 25 (53%) had a high risk of chemotherapy toxicity **(** Table 2). CARG toxicity scores were useful in predicting the presence of at least one grade 3 or higher adverse event (Figure 3). The majority (74%) of participants experienced a grade 1 or two treatment-related adverse events and 10 (20%) of participants experienced a grade 3 or higher adverse event. Participants who scored ≤7 on the CARG toxicity assessment tool (47%) had three participants with a ≥grade 3 while those that scored ≥8 (53%) included seven participants that experienced a ≥grade 3 (70%) that occurred during this study. Patient-reported outcomes measuring physical function (IADLs, PROMIS 10, MOS) among this cohort did not show high levels of disability while the SPPB suggests the presence of physical impairment (median: 9) and 19 participants (38%) had TUG scores that indicated a mild risk for falls. At baseline, participants showed a mild to moderate concern for cognitive impairment (BOMC (Tuch et al., 2021) median: 6) (Table 2)**.**

### 3.4 Relationship between geriatric assessments and molecular biomarkers

**FIGURE 4:**
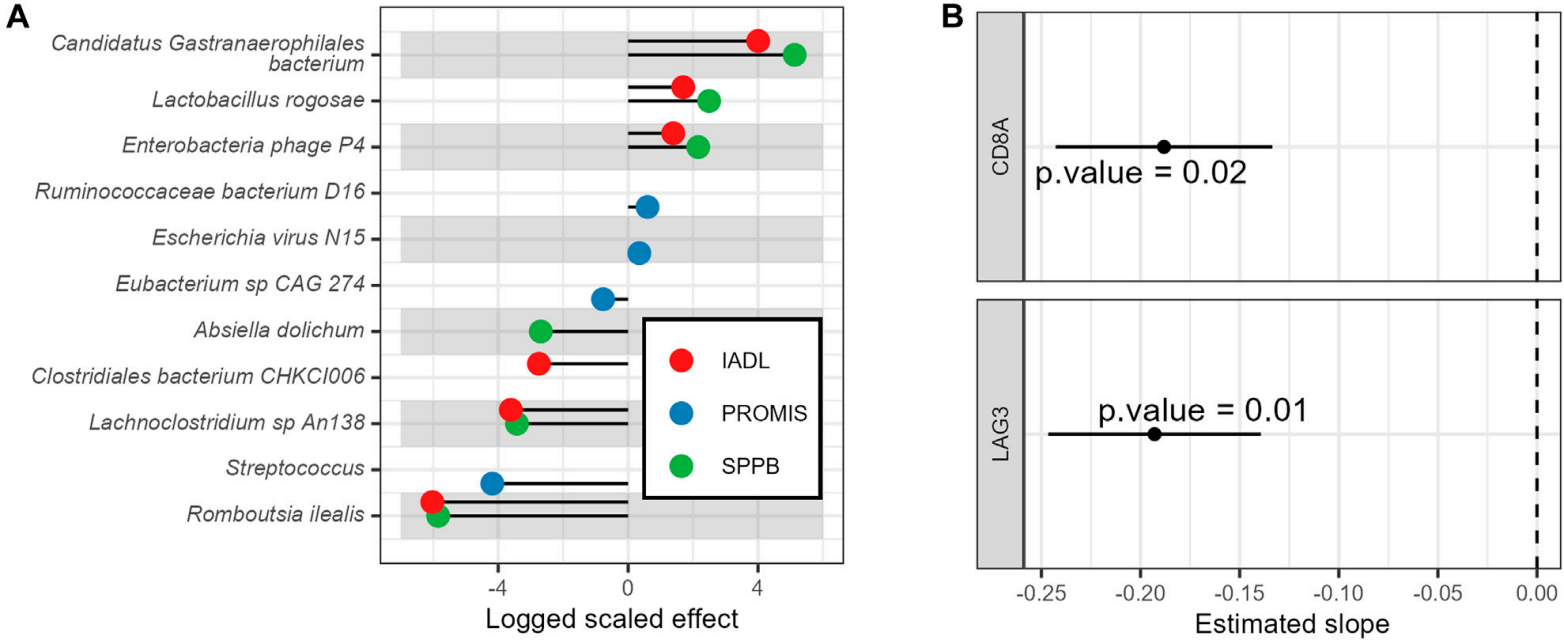
Associations of baseline vs. longitudinal geriatric assessments with molecular biomarkers. **(A)** Longitudinal associations of geriatric assessments with gut microbe relative abundances and **(B)** Longitudinal associations of IADL with T-cell gene expression. Displayed are the model’s estimated slopes with 95% confidence interval between genes and IADL.

Changes in GA tools over time were associated with changes in molecular features, including the gut microbiome and T-cell senescence markers. For example, *Lactobacillus rogosae, was associated* with increased (improving) IADL, QoL, and SPPB scores (Figure 4A; complete results with all *p*-values corrected for multiple hypothesis testing appear in Supplementary Table S1). In contrast, the microbes *Romboutsia ilealis*, *Streptococcus*, and *Lachnoclostridium sp An138* were associated with decreased (worsening) IADL, QoL, and SPPB scores over time (Figure 4A). Similarly, T-cell *lag3* and *cd8a* RNA expression over time were associated with decreased IADL scores (Figure 4B; complete results with all *p*-values corrected for multiple hypothesis testing appear in Supplementary Table S2, with results of an additional sensitivity analysis of these results using Spearman correlations appearing in Supplementary Table S3 and the full dataset appearing in Supplementary Table S4).

## 4 Discussion

This study builds upon prior research supporting the utility of the CARG CGA in patients with lung cancer and describes the feasibility of longitudinal data and biospecimen capture in this patient population. Our comprehensive study design captured the GA longitudinally, included patients receiving the newest lung cancer treatments, and collected blood and stool samples for correlative biomarker analysis, resulting in a rich dataset. At baseline, patients reported minimal functional impairment and relatively high social support and exhibited normal levels of mobility as measured by the TUG. However, we uncovered mild to moderate levels of cognitive impairment using the BOMC and physical function impairment using the SPPB.

This study demonstrates the feasibility of instituting the CARG CGA longitudinally among older adults with lung cancer, with ≥50% of participants completing the surveys at each time point. The CARG toxicity calculator may also be useful in predicting treatment toxicity risk among older adults with NSCLC receiving newer treatments. We suspect withdrawal due to study burden (increased visit time and survey fatigue) was largely a result of the inclusion of recently diagnosed patients who were beginning treatment, contributing to general overwhelming feelings upon arrival at study visit 1.

We gained valuable insights into the preferred timing and modality of longitudinal GAs among older adults diagnosed with lung cancer. Due to patient status decline and death, we amended this study to include an additional GA at study visit 3, or roughly 6 months into treatment. We hypothesize that more frequent assessments would improve follow-up data capture for longitudinal data collection among participants with advanced disease. Additionally, we hypothesized that offering patients the option to complete study surveys virtually through a secure email link, allowing them to complete them independently rather than during their oncology visits, may mitigate survey fatigue and, ultimately, study dropout rates.

Prior research using GAs for various cancer types lacked physical performance metrics and relied solely on blood-based biomarkers (Antonio et al., 2018; Honecker et al., 2018; Loh et al., 2020). Furthermore, most current literature on older adults only included patients 70 or older, overlooking potential differences in chronologic *versus* physiologic aging (Schulkes et al., 2016). Physiologic aging is a complex process dependent on many biological and sociodemographic factors (Dziechciaz and Filip, 2014). To better account for biological aging-associated changes, this study included adults ≥60 years of age.

One limitation of this study is the dropout rate, leading to missing longitudinal data for many participants. The study follow-up length likely contributed to the high attrition rate due to death, as those who died while on the study had more advanced stages of the disease and thus longer treatment plans, permitting study enrollment for up to 12 months. In general, participants on shorter treatment plans were only asked to participate in the baseline and EOT visits and likely presented with early-stage disease with a lower risk of adverse events and disease complications. Another limitation of this data is the sample homogeneity. Thus, our findings may only be applicable to sociodemographic groups within this single-site center. While a proportion of participants declined or had poor adherence to stool sample collection, we gained valuable insights and strategies to enhance future gut microbiome studies in older adults with lung cancer. Patients who provided a baseline stool sample were likelier to submit a follow-up sample than those who did not participate at baseline. We hypothesize that reminding participants *via* phone 24–48 h before their clinic visit to collect stool samples, with incentives offered, could improve adherence and overall acceptability of sample procurement.

Future research should investigate whether modifying these biomarkers can impact treatment outcomes and establish causality in these associations. One upcoming randomized clinical trial (Geriatric Assessment and Management for Older Adults with NSCLC Receiving Chemotherapy Radiation Therapy (GAM-CRT (NCT06139627)), open to accrual, will examine the association between geriatric assessments and physician recommendations with grade 3–5 treatment toxicities. The GAM-CRT trial will continue to explore the relationship between microbial diversity and blood-based biomarkers with treatment-related toxicity and disease response. Additionally, the collection of PROs could be integrated into the electronic medical record (EMR) for improved care pathway implementation. While our institution has since incorporated PROs including the Cancer and Aging Research Group Chemo -Toxicity Calculator, The Geriatric 8 Screener, Generalized Anxiety Disorder −7 (GAD-7), Patient Health Questionnaire–9 (PHQ-9), and the European Quality of Life–5 Dimension (EQ-5D-5L) into the EMR, there are no current care pathways. This is an area for future development in which the GAM-CRT trial will help to distinguish these optimal recommendations such as referrals and other supportive care interventions.

In conclusion, our findings suggest that the CARG CGA can be successfully administered to newly diagnosed older adults with lung cancer prior to treatment initiation and at follow-up. However, assessments should be conducted at intervals of no longer than 3 months to improve longitudinal data collection. We found that baseline and longitudinal blood and stool samples were attainable and could be assessed for correlation with physical function measures, including the SPPB, and PROs, such as IADL impairment and quality of life. We plan to continue evaluating multidisciplinary GAs and their associations with correlative biomarkers and clinical outcomes to refine the gold standard for geriatric assessment procedures. Ultimately, the knowledge gained will improve therapeutic decision-making, risk assessment, patient prioritization, and quality of life for older adults with lung cancer.

## Data Availability

The data and code to regenerate all analyses is publicly available in the http://github.com/spakowiczlab/fitness repository, at DOI 10.5281/zenodo.11200745.

## Supplementary material

SUPPLEMENTARY TABLE S1 Longitudinal Models for Microbes and Geriatric Assessments 574.

SUPPLEMENTARY TABLE S2 Longitudinal Models for Nanostring Results and Geriatric Assessments.

SUPPLEMENTARY TABLE S3 Longitudinal Spearman Correlations for Select Microbes, Nanostring Results, and Geriatric Assessments.

SUPPLEMENTARY TABLE S4 Complete Longitudinal Clinical Source Data.
